# Corporate power and global value chains: current approaches for conceptualizing the power of multinationals

**DOI:** 10.1007/s43253-024-00121-5

**Published:** 2024-06-19

**Authors:** Jakob Kapeller, Claudius Gräbner-Radkowitsch, Anna Hornykewycz

**Affiliations:** 1 Institute for Socio-Economics, University of Duisburg-Essen, Duisburg, Germany; 2 Institute for Comprehensive Analysis of the Economy (ICAE), Johannes Kepler University Linz, Linz, Austria; 3 Department of Pluralist Economics, Europa-University Flensburg, Flensburg, Germany

**Keywords:** Power, Corporations, Corporate power, Globalization, Networks, Race for the best location, B50, F55, F60, F63, P12

## Abstract

The influential position of multinational corporations in the global economy of the twenty-first century is a particularly controversial and timely subject. This paper aims to improve our understanding of this phenomenon by focusing on one particular aspect of it: corporate power. To this end, it first puts forth a number of conceptual clarifications that help to distinguish different kinds of power and the distinct analytical levels at which power is executed. It then focuses on corporate power and studies it against the backdrop of the development of global value chains. The aim of this analysis is twofold: firstly, to review the variety of analytical tools and ontological perspectives that coin current research on corporate power, and secondly, to discuss the causes and practical consequences of asymmetrical power constellations among corporations and between corporations and other actors. This discussion is meant to facilitate a better alignment of applied research on corporate power with pluralist approaches towards rethinking economics.

## Introduction

“There has never been any doubt that whoever controls access to the citizens’ livelihood exercises political control.” (Drucker 1962)

In today’s globalized economies, multinational corporations (MNCs) are often seen as embodying the archetype of corporate power. Due to their multinational structure, these corporations enjoy greater opportunities for action, thus enabling them to go beyond more traditional understandings of corporate power, which have focused primarily on issues of market power and business strategy, price- and wage-setting decisions, or questions of R &D and product design (for earlier heterodox analyses that consider corporate power more broadly but without an explicit focus on multinationals, see, e.g., Veblen (2022); Robinson (1962), and Galbraith (1998), respectively). Rather, due to their exceptional size and multinational structure, MNCs control significant shares of global production and distribution. Their strategic decisions—e.g., on where to invest or pay taxes—directly impact local and regional economic developments. This motivates the investigation of the nature, sources, and consequences of their power.

From a historical perspective, internationally operating corporations are not a new phenomenon. They have been understood as relevant and influential institutions since, at the latest, the “first globalization,” a period from around 1870 to 1914 which followed the industrial revolution, and that was characterized by a significant increase in international economic integration (Fitzgerald 2015, Chapter 2). Their origins, however, extend even further back into the past, at least to the *commercial age*, the pre-industrial era of international sea- and long-distance trade (approximately 1500–1800 according to Allen (2011)). This era was pivotal for shaping the path-dependent long-term growth trends associated with the so-called *Great Divergence*, and the socio-economic structures that were formed in this era are recognized as enduring and relevant by several heterodox approaches, such as the world-systems analysis (Frame 2022; Arrighi and Silver 1999; Wallerstein 2011, e.g.) or dependency theory (Kvangraven 2020, e.g.), as well as broader movements such as degrowth (Gräbner-Radkowitsch and Strunk 2023, e.g.).

And indeed, multinational firms have played an important role in shaping and preserving this path dependency. Early examples, such as the East India Companies, which were founded in 1600 (Britain) and 1602 (the Netherlands), illustrate this historical relevance of MNCs from a *longue durree* perspective (see, e.g., Braudel and Wallerstein (2009)) and indicate the intrinsic connection of such a historical perspective to a postcolonial perspective on the relevance of MNCs, global value chains, and socio-economic development (see, e.g., Alami et al. 2023, and below). However, despite this historical continuity, recent decades showed a qualitative shift in the role of corporations both within society and globally.

The liberalization of capital markets following the collapse of the Bretton-Woods regime in 1973 is considered a central factor in this context (Eichengreen 2010) as it allowed for a hitherto unseen expansion of international financial capital, which made acquisitions of property in the Global South by MNCs much easier. Hence, this shift contributed to an expansion of the scope and sphere of influence of MNCs and significantly strengthened their negotiating position compared to other actors—such as workers, governments, or smaller firms (Rothschild 2005). It is this development that caused a marked increase in the significance (and power) of MNCs in recent decades, and that motivates this paper.

Alongside or in parallel to the collapse of the Bretton-Woods regime and the abolishing of regulations, another development essential for understanding the particularly privileged position of MNCs in today’s global capitalism is the increasing importance, depth, and differentiation of global value chains (GVCs). As MNCs are typically said to create, shape, and govern (certain segments of) GVCs, the increasing importance of the latter is both a prerequisite for and a consequence of the internationalization of firms. This mutually dependent relationship indicates a co-evolution between the increase in power of MNCs and the growing internationalization of production processes and value chains. It also implies that GVCs play a central role in understanding why MNCs agglomerate power and which strategies are used to increase, execute, and stabilize this power. Against this backdrop, a closer examination of corporate power within the context of GVCs seems to be attractive.

To provide a starting point for such an examination, this article sets out to do two things: (1) to review the variety of analytical tools and ontological perspectives that coin current research on corporate power and (2) to discuss the causes and practical consequences of asymmetrical power constellations among firms as well as between MNCs and other actors, like governments or unions.

By doing so, this article also tries to contribute to a more general agenda, namely to facilitate a better alignment of different strands of literature on corporate power, classic theories of power from heterodox economics, recent contributions from postcolonial studies (Go, 2016), and pluralist approaches towards rethinking economics. Such an alignment seems attractive, because several core commonalities among these approaches are immediately evident, such as a focus on relations and networks and the consideration of distributional issues and associated power asymmetries. At the same time, any attempt to triangulate concepts from hitherto separated domains requires a common meta-theoretical framework that supplies the glue for the integration, usually in the form of a shared language, a consistent ontology, and a common epistemology. The framework we use here is that of Mario Bunge’s “systemism” (see Bunge, 1996; Bunge, 2004), which has been argued to provide a useful framework for heterodox economics in general (Grabner and Kapeller 2017). The fundamental ontological object in Bunge’s systemism is the “system.” More precisely, according to Bunge, every entity should be considered a system or a part of one. Systems, in this sense, are constituted by three core elements—(1) basic parts, (2) an environment, and (3) a relational structure—that eventually bring forth (new) mechanisms (Bunge 2004). As will be shown below, this very general blueprint will prove useful in integrating contributions on corporate power and GVCs from different intellectual domains, which initially all come with their slightly distinct terms.

The paper is outlined as follows. First, Sect. 2 introduces basic concepts and definitions, whereas in Sect. 3, we discuss central theories of (corporate) power and develop an analytical heuristic that will enable our further analysis of corporate power in GVCs. In Sects. 4 and 5, we then introduce different ways to explain (patterns of) corporate power and illustrate the related diversity of approaches towards analyzing corporate power, which we group and summarize with respect to different levels of analysis.

In Sect. 6, we conclude the paper by taking the relation between increasing corporate power and rising inequality as an example to illuminate some global consequences associated with the historical rise of MNCs in the age of (hyper-)globalization.

## A systemist approach to corporate power: core terms and concepts

According to conventional definitions, a *(global) value chain* consists of labor and production steps that build upon another to culminate in a consumer-ready product (Hopkins and Wallerstein 1986). MNCs are understood as transnationally operating entities that are engaged in multiple value chains *(horizontal integration)* and/or various stages within the same value chain *(vertical integration)*. Both phenomena can be thought of (or represented) as overlapping networks: GVCs arise from a network of interconnected yet locally separated labor and production activities, while MNCs typically cover a specific segment of this network of GVCs. Applied research that investigates the constitution and change of specific GVCs can, hence, be differentiated from research that focuses more specifically on how certain (segments of) GVCs are governed by specific MNCs to create so-called *global production networks* (GPN) (Neilson et al. 2014).

This way of conceptualizing MNCs and their associated GVCs aligns nicely with Bunge’s systemism and its definition of system described above since it is straightforward to consider MNCs and the associated GVCs and GPNs as social systems. The consideration of relational structures in systemism proves to be especially useful in our context as it provides a clear link to established conceptualizations of MNCs and GVCs, which are often represented as (overlapping) networks, e.g., when Dicken (2015, p. 130) defines MNCs as “networks within networks,” Here, a multinational corporation is understood as a network of firms, locations, or contractually bound partner firms that is, in turn, embedded in the broader network of GVCs. Also, treating corporations and GVCs as social (sub-)systems has the advantage to avoid conflating them with the associated networks—it makes explicit that such networks are coined and partially even governed by local actors and subjected to environmental influences, such as changes in public opinion or international regulations.

**Fig. 1:**
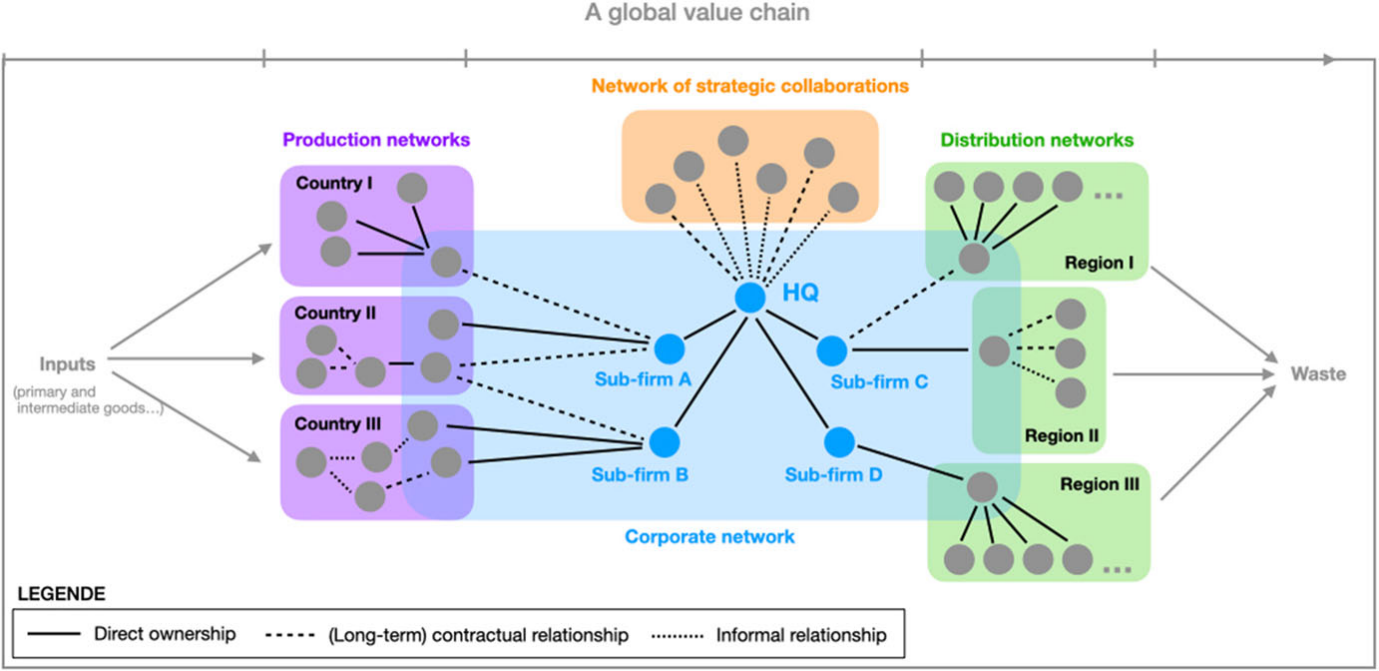
The positioning of a multinational corporation within transnational value chains, inspired by Dicken (2015, p. 131)

Figure 1 illustrates this perspective and depicts the typical positioning of a MNC within GVC. It also illustrates the layered ontology of Bunge’s systemism as the entire value chain is considered to be a system which has four interconnected subsystems: (1) the core corporate network around the headquarter firm (HQ) as a core actor, (2) a network of strategic collaborations associated directly with this core actor, (3) a set of production networks, and (4) a set of distribution networks. Each of these parts can be considered a system in itself, with an associated relational structure between its parts, and specific mechanisms operating within and between these subsystems. This means that each individual actor (here: usually firms) is to be considered a single part of the system, which is embedded in a particular position in the overall value chain.^1^ Due to this close entanglement, we will use the terms networks and systems largely synonymous in what follows.

Using this systemist approach to understand the role of MNCs within GVCs is also helpful in highlighting several other characteristics of MNCs and GVCs. First, the precise relationship between the individual actors can be quite heterogeneous: each actor is embedded in different subsystems within the overall GVC and can be related to the core firm in various formal or informal ways, including direct ownership relationships, long-term formal contracts, or merely informal cooperation practices. From this, it follows that each level and subsystem might also be affected by different mechanisms, and the systemist vocabulary makes it easy to explicate this. Second, a systemist approach also makes it easier to highlight the importance of how the segment of the value chain governed by an MNC is embedded in a fragmented environment consisting of different geographical locations and governed by the fact that the corporate headquarters has privileged access to actors that influence institutional conditions. Third, it allows to study the ability of MNCs to form, stabilize, and exploit specialized networks based on targeted functional differentiation, that is, to create a global production network (GPN). For example, it can be used to illustrate the impact of corporate power on the geography of GPNs, which typically consist of both a *production* network in the narrow sense and a corresponding *distribution* network. Through such a distribution network, corporations aim to cover as large an area as possible to ensure a wide availability of the respective intermediate or final products that are eventually sold to external parties. In contrast, production networks are much more punctually present and locally rooted. They concentrate especially in industrial clusters, coastal urban centers, and special economic zones, occupying a much smaller space than distribution networks (Dicken 2015). As a consequence, actors in distribution systems typically complement each other on a spatial level, while actors in production systems find themselves in competitive setups with higher probability—regardless of the spatial dimension.

**Table 1:** Analytical categories of corporate power

**Dimension**	**Examples for entities considered**	**Examples for analytical questions**
Environment	Entities outside of the power relationship of interest	Which factors constrain the behavior of the actors?
	The institutional and natural environment in which the actors operate	What are the social rules that structure the interaction and exercise of power?
Components	Number, identity, and motivation of involved corporations	Who are the corporations involved, what motivates them and what are their reservation options?
Network structure	Relationships between the components of the system	Which corporations occupy powerful positions and why?
		How are reservation options affected by the corporation’s position in the network?
Mechanisms	Means for enforcement of will	How do actors exercise power? How is their power being challenged?

The analytical language is consistent with Bunge’s systemism and its corresponding CESM model (Bunge, 2004). Columns two and three only contain examples and should not be considered to provide a complete of everything that could be studied on this level

This theoretical framework suggests that various ontological levels are of potential interest if one studies the subject of corporate power. As indicated above, to achieve our goal of triangulating theories and concepts from different silos of previous research, we use the language and theory of “systemism” to map the contributions of existing studies onto a coherent framework. Table 1 provides a summary and aligns the different levels with the essential analytical categories of Bunge’s systemism.

More precisely, Bunge (2004) suggests that to describe any system, one would need to describe (i) the set of its essential parts (its “composition”), (ii) the environmental items that affect or are affected by the system, (iii) the network structure that holds the components of the systems together, and (iv) the characteristic processes or mechanisms operating within the system. In Table 1, these dimensions are listed in the first column. The second column contains some examples for entities to which these dimensions refer in practice. Column three then gives examples for typical analytical questions posed on this level.

## Theories of corporate power

“[Economics] as a separate science is unrealistic. [...] It is one element – a very important element, it is true – in a wider study, the science of power.” Russell (2004)

Power is a central and yet contested concept in the social and economic sciences. In this contribution, the focus will be on theoretical aspects of power that can be operationalized clearly. Therefore, in this section, we delineate manageable concepts, which facilitate the analysis of concrete power relations and their effects.

### Classic notions of power: direct and indirect power

To get a first grasp of the concept of power, it is helpful to distinguish between *direct* and *indirect* forms of power exertion. *Direct* forms of power exertion typically manifest in a manner where the involved actors and their mutual connections, conflicts, and dependencies can be identified and named relatively clearly. Consequently, the relevant power relations are usually directly observable, and, in many cases, the affected actors are also consciously aware of them. In contrast, *indirect* forms of power exertion refer to situations in which individual actors manage to shape basic ideas and motives of social, economic, and political action with the effect that their interests are (even) better served. This can be due to conscious strategy, structural asymmetries, or coincidental circumstances. Typical examples for indirect forms of power exertion comprise standardization, i.e., technical restrictions that need to be taken into account, or hegemony, i.e., prevailing political ideas (see Graz (2019) for a more comprehensive treatment on standards). Notwithstanding the fact that standards—the result of power exertion in these contexts—are often formalized and transparent, the underlying power exertion as such is often not directly identifiable but remains rather vague (Dallas et al., 2019; Graz, 2019). Consequently, it often goes unnoticed by the affected actors. As scholars like Lukes (2005) or Han (2018) argue, this is precisely wherein the core strength of *indirect* power exertion lies.

Direct power relations are easier to describe, and their discussion has a long tradition in the social sciences (e.g., Weber (1980); French (1956); Dahl (1957)). Following this tradition and drawing on Weber (1980, p.28), a specific direct power relation can be defined as follows:

“Power of A over B means that within the social relationship to B, A can enforce their own will even against the resistance of B.”

In this perspective, the specific configuration of a respective power relation depends significantly on the means available to both actors. For A, the question is by which means to enforce their will or interests, while for B, reservation options matter. Reservation options (also: *exit options*) can be understood as the next-best alternative B has relative to complying to the demands of A.

Both concepts—means and reservation options—are kept abstract so that they can take various forms in practice and need to be interpreted differently depending on the situation considered. Generally, means encompass categories such as threat, monetary influence, position, sanction, violence, cunning, social relationships, negotiation, or contract (as already pointed out by Machiavelli (2003)). While the categories considered are similar for reservation options, the focus here is on B’s *second-best option* (i.e., their *exit option*). Theories of continuous power relations, therefore, often establish a direct connection between the means of A and the reservation options of B.

Most arguments on power in heterodox economics articulate specific cases that resemble this overall pattern. An example is given by Marx’s framework, where workers are forced to sell their labor power because they lack ownership of the means of production, which, in turn, constitute the central source of the power of capital. To bring this example in line with our technical terminology, the means of power are constituted by private property, and hence, workers find themselves without a viable reservation option as they lack such property. The underlying “social relation” between labor and capital thus constitutes a long-term asymmetric power relation (see Marx and Engels (1983), pp. 822-839). A similar reasoning can be employed to cover other classic conceptions of power and/or power asymmetries in heterodox economics, like the gendered power asymmetry associated with conventional forms of social reproduction (see, e.g., Bhattacharya (2017)), power asymmetries in international trade due to distinct technological capabilities (Sapsford et al. 1992; Ocampo 2022), or the power asymmetry associated with private monopolies in markets for basic goods with a low elasticity of demand, like food, water, or electricity (Collective 2018).

When analyzing power relations, it seems a helpful first step to specify both the involved actors and the two categories of means and reservation options as precisely as possible. Such an application is most straightforward when analyzing dyadic and direct power relationships. In the next section, we will discuss how the classical power formula can be expanded or adapted to also capture more complex power relationships.

### The classical power formula as a heuristic for situations with many actors and indirect power relations

While the classical power formula provides a suitable definition of power and an initial description of specific (especially *direct*) power constellations, its scope remains restricted in at least two dimensions. First, it is debatable whether and to what extent it can be used to describe *indirect* power relations. Second, the power formula has its focus on specific social relationships that involve two actors, A and B. In order to apply the formula to networks or social structures (which will often seem necessary if one wishes to study corporate power), it requires modification. In other words, the classical power formula primarily serves as a first vantage point that needs to be operationalized with careful consideration of the specifics of the situation under study. Yet, with its focus on means and reservation options, it provides a general framework that can in principle be applied to various social contexts.

This framework can be employed to derive case-specific variations of the classical power formula. An example is provided by the economic analysis of competitive situations such as labor market competition or regulatory races for the best location: In these cases, the agency of actors is indirectly proportional to the competitive pressure they face. That is, when the competitive pressure is high, actors are largely powerless (see, e.g., Weber (2015, pp. 43–44), but also Glotzl et al. (2019)). Conversely, and in analogy to the classic power formula, actors are considered powerful when they can employ competition as a means to derive direct benefits—which, in most cases, will imply that they reap benefit *from the competition of others*. Similarly, actors can be argued to be in a powerful position, when they are able to successfully evade competitive pressures, that would make them vulnerable to exploitation by third parties.

In this sense, the above formula could be reformulated from an economic perspective as follows:

“Power of A increases with the number of agents competing for satisfying A’s needs and diminishes with the number of agents A has to compete with.”

Examples of such situations can be found in a wide range of social contexts. Notably, this version of classic power formula not only builds a conceptual bridge to the classic notions of market power (monopoly, oligopoly, monopsony...) prevalent across different economic traditions, but also lends itself to the study of corporate power including the specific case of MNCs (see also Sect. 5.3).

An obvious example relates to those GVCs, where few potential buyers make their potential suppliers compete with each other, another to the globalized race to the best location, e.g., when states “commercialize sovereignity” (Palan 2002) to compete for investments from multinational corporations (see Rodrik 2011), but they can also be found in different contexts.^2^

This reformulation of the power formula implies a *multitude of intertwined relationships* rather than a specific relationship between two actors, and hence, it scales the classic power formula in a way that lends itself well to many economically relevant constellations of power asymmetries; moreover, it also captures core aspects of the power of MNCs.

By emphasizing the relative positioning of A, the adapted power formula draws attention to the qualitative properties of the underlying relational setup. It is sensitive to the fact that the relationship between different suppliers is structurally different from that between suppliers and buyers. However, this notion of power quickly becomes ambiguous if the relational structure underlying the power dynamic is not made explicit. To tackle this problem and explicate this structure in the context of value chain analysis, using networks as a method of representation presents itself as a promising methodological approach (more on this in Sect. 5.2).

Eventually, such representations of direct power also provide a route towards the quantification of economic forms of power. Typically, such considerations boil down to questions related to the *allocation* of production factors (like primary resources, land, technology, capital, or (labor) time) or institutional positions (along established hierarchies and stratification dimensions) on the input side. On the side of outcomes, the *distribution* of certain variables, like income, leisure time, or exposure to hazards, is considered a yardstick to judge the impact of power asymmetries in such a context.^3^

While these examples indicated how to adapt the traditional, dyadic formulation of the power formula to cover a greater number of actors, it remains a challenge to identify and describe indirect power constellations in practice. This, of course, does not imply that indirect forms of power are less relevant—quite the opposite. According to Lukes (2005), it is the unconscious, indirect power relations that are particularly effective and relevant. The emergence of indirect power relations usually extends over a longer period, as the underlying routines, structures, and ideas can only develop over time. A prominent example of indirect power constellations with regard to corporate behavior is the issue of standard setting, i.e., the establishment of dominant designs and associated technical or social norms (Graz 2019): Power that arises from standard setting becomes particularly evident in products whose utility depends on the widespread adoption of the product, as seen in operating systems such as Microsoft Windows or GNU Linux, programming languages such as Java or Python, or communication platforms such as Skype or Zoom.

The dominance of these standards is mostly due to the positive feedback that is associated with their use: as Dobusch and Kapeller (2013) note, whenever “positive feedback is at work and a series of competing and incommensurable social standards *x* are available, then one of these standards will tend to dominate.” The sources of the positive feedback can, however, be quite diverse. On the one hand, there are sources that relate to the coordination problems underlying the use of the respective goods and services: for instance, when the number of people using a particular programming language becomes larger, it becomes more attractive to newcomers (“external” positive feedback according to Dobusch and Kapeller (2013)). Similarly, whenever an individual actor decided to use a particular production technique and learning effects are relevant, then sticking to the same technology becomes more attractive (“internal” positive feedback according to Dobusch and Kapeller 2013).

On the other hand, there are also situations where the emergence of standards is governed by corresponding actors. The *International Accounting Standards Board* (IASB), for instance, is a private body that, together with the *International Financial Reporting Standards* (IFRS) Foundatation, develops, audits, and distributes standards for accounting. Here, the stability of a standard is curated explicitly by a private actor. A similar, yet even broader example is the *International Organization for Standardization* (ISO).

No matter whether standards emerge rather spontaneously as a solution to an underlying coordination problem, or because they are governed by a standard developing organization, there is always the result of positive feedback, leading to significant *switching costs* and thus endowing providers and early adopters with considerable power gains: the reservation options of their customers become less and less attractive the more users are following the dominant standard (for more details, see, e.g., Heinrich 2014, 2018). Here, the successful setting of standards usually comes with a self-reinforcing dynamics: while the successful setting of standards is, of course, facilitated by an already existing amount of power Commons 1934; Graz 2019), the key point is that once a standard is set, the power of the standard setter gets further stabilized or increased.

In such cases, actors are not necessarily aware of the actual exercise of power: once legal, institutional, cultural, or technical standards are established, they are often taken for granted. In this situation, we may find ourselves in a scenario described by Lukes (2005, pp. 28) as the “supreme and most insidious exercise of power,” precisely because the status quo is no longer questioned by the subordinate actors. A commonality of such standards—independently of whether they emergence spontaneously or are deployed strategically—is that they shape and restructure the networks underlying the exertion of direct power. Hence, such indirect forms of power are often consciously exploited to shape the long-term playing field in one’s favor allowing for stabilizing or extending existing power asymmetries, from which powerful actors profit in the short run. Therefore, in the analysis of indirect power exertion, it seems necessary to pay special attention to the question of awareness of power relations among the involved actors.

## Sources of corporate power

The classical power formula is somewhat abstract as it does not deal with the specific origin or cause of existing power relations. These essential aspects must be supplied externally.^4^ Nevertheless, the formula implicitly points to a crucial source of power—namely, the position of an actor within a larger network of relationships. A typical example that illustrates the power gain associated with a dominant network position is the—nowadays most often digital—platform economy (Elmer and Hofmann 2016; Grabner and Heinrich 2017). Platforms are a new business model that reach beyond traditional conceptions of a firm by providing an infrastructure that allows several potential groups of users, such as providers and customers to connect (Srnicek 2017).^5^ Examples include *Amazon Marketplace* or Alibaba (Siu 2023), which connect providers and demanders of various products, and the video platform *YouTube*, which provides producers and consumers of videos with a communication platform. However, examples extend beyond the digital economy. They encompass credit card companies that connect buyers and sellers (e.g., VISA), stock market companies (e.g., XETRA), and large retailers and specialty stores (such as Walmart or Costco), which also function as powerful platform companies. Such platforms thus occupy a central position and act as gatekeepers for other market participants, often allowing them to set increasingly unfavorable business conditions and prices for other market participants. Due to their lack of attractive reservation options, the latter are usually relatively defenseless against this exercise of power. The importance of platforms has drastically increased in recent decades, and they have received more attention in economic research. In particular, trends such as the increasing precariousness of working conditions, which is associated with platforms’ tendency to outsource a multitude of tasks, or the accumulation of large amounts of personal data have raised concerns in the past (see, e.g., Srnicek 2017; Langley and Leyshon 2017; Arrieta-Ibarra et al. 2018). Some scholars argue that their central network position will gain influence in the economic system in such a way that the net impact of other sources of power will become negligible (for further literature on global economic organization and accumulation in this context, see, e.g., Montalban et al. 2019; Bratton 2016; Likavčan and Scholz-Wäckerle 2022).

However, in many cases, an advantageous network position is a consequence of other factors that can, in turn, be identified as significant sources of power. A main source of power in economic history is the legal institution of private property rights of resources, land, means of production and—especially in newer developments—ideas.^6^

Private property rights typically allow the actor who holds them to extract profits, and, on top of that, different kinds of rents which, in turn, can also be seen as an emergent outcome of prevailing power constellations. As the existence of a rent signifies income *beyond* conventional profits, such rents are closely associated with the pre-eminent position obtained by MNCs. While all kinds of rents have in common that they are both a source and result of corporate power, there are qualitative differences between them worth mentioning: first, the conception of a *ground rent* derives from a power relationship between a landlord and a tenant (Marx and Engels 1983, Parts 5 and 6), where the former enjoys the opportunity to extract the rent that the land yields from the tenant. The often violent conflicts over the establishment of private property rights in the Global South and the related enclosures in the context of land grabbing by multinational firms today (Rulli et al. 2013; Margulis et al. 2013), or the historical enclosures in the UK that facilitated the industrial revolution by “creating” the “free workers” illustrate how entangled power relations in these contexts often are Marx (2021, Chapter 24). Thereby, the notion of a *ground rent* explicitly incorporates a notion of heterogeneity as it is based on the observation that the returns from extraction may differ substantially across different segments of land and, hence, the notion of a rent is tied to the idea that some agents possess more rewarding property rights than others. This aspect also endowed the concept of a *ground rent*, which originally referred to differences in agricultural productivity only (Ricardo 1955, Volume 4, 1–42), with a certain generality, that enabled a broader and more inclusive use of the term (which is sometimes also labeled as *property rent*).

Second, and in contrast, *monopoly rents* are extracted by firms that have gained control over a market, thus putting them in a position to limit access for newcomers (Davis et al. 2018; Bowles et al. 2017). There is a long-standing interest among economists of different persuasions to assess the impact of market power, which allows for bridging traditional approaches from economics and political economy with more explicit research agendas focusing on the power of MNCs. A special case of a monopoly rent is *innovation rents*, that can be extracted when a firm attains a monopoly position by introducing a new product, process, or business model (Bowles et al. 2017). This kind of innovation rent usually diminishes in the short- or mid-term when other firms enter the market for the innovated good. With regard to market power more generally, there exist further, specific contributions in the respective literature that aim to pin down the exact source of market power more concretely. A prominent example is given by *information rents*, where market power is not proxied by size or market share, but, rather, by assessing the distribution of information across market participants and the economic consequences arising from these potentially asymmetric distributions (see, e.g., Myers 2020; Rotta 2022).

Third, the last decades have shown an increased relevance of knowledge-intensive technologies. While knowledge is, in principle, non-exclusive and a development towards more knowledge-intense technologies might suggest a redistribution of power towards smaller firms, the legal enforcement of intellectual property rights from the 1990s onward created the possibility of *intellectual rent* extraction, which, in turn, mimics the general logic of ground rents for the case of immaterial goods. Therefore, other than traditional innovation rents, intellectual rents can, via their focus on intangible assets, extend their temporality beyond the mid-term. Due to its recent relevance, this new mechanism of the accumulation of corporate power has been dubbed “intellectual monopoly capitalism” (see, e.g., Rikap 2021; Pagano 2014; Durand and Milberg 2020). Lastly, financial rents can be extracted by access to favorable financial and tax conditions. This type of rent is especially relevant in the case of intellectual monopolies that are more flexible to move their organization geographically (Rikap 2021).

Another important source of corporate power is found in a high number of potential reservation options. Via the possibility of outsourcing, i.e., the geographical relocation of activities to pursue a cost-minimizing strategy, reservation options allow for flexibility of global corporate activity (Milberg and Winkler 2013). In this sense, outsourcing provides exit options relative to a series of diverse constraints, like high wage demands, labor law, environmental regulations or tax burdens. As a result, the use of such reservation options shapes the establishment and structure of GVC. A key notion here is that outsourcing allows corporations to exploit international differences in production environments and legal regulations to its own advantage. This specific form of innovation, that is based on finding new opportunities for bypassing existing legal and social obligations in turn, enables MNCs to, at least partially, sidestep existing regulations (Kapeller et al. 2016; Ruggie 2017). Outsourcing however is not only a strategy fit to escape local constraints but also a mechanism that helps reinforce existing global power relations in pressuring regions of the Global South to maintain regulations that, although advantageous for MNCs, offer unfavorable conditions for the local population, including lower wages and worse work conditions (Suwandi 2019).

Yet although MNCs are indeed powerful actors, there are also factors that can limit and restrict corporate power. Limiting factors include political interventions and regulation by state authorities, civil society initiatives opposing the effects of corporate power at different levels, and, not least, competition between various MNCs, which can significantly limit the power of individual actors. This last aspect implies that tendencies and strategies dedicated to the agglomeration of further power can, in view of the fact that competitive pressures limit a corporation’s power, also be the result of a mere reactive behavior aiming to secure an actor’s current power position, which is seen as inherently contested (Shaikh 2016). In this view, outsourcing behavior can also be rationalized as a consequence of existing competitive pressure among corporations. This points to the importance of relative perspectives: while outsourcing surely constitutes a central source of corporate power from an analyst’s perspective, it may present itself as the only viable alternative from the perspective of the individual company or manager, whose perspective is coined by the aim to, at least, stabilize the current relative position of some company.

“If you are the CEO of a major firm and can increase your profits by offshoring, why not do it? If you don’t relocate some of your operations and your competitors do – and increase their own profits in the process – you are unlikely to last.” (Gomory 2009, cited in Milberg and Winkler 2013, p.25)

The following chapter attempts to provide a more detailed and precise picture of different forms of corporate power mainly by identifying different levels and providing concrete examples trying to illustrate the manifold facets corporate power may take. These manifold forms have, in turn, an impact on the appropriate way of describing and analyzing instances of corporate power, especially in the context of MNCs.

## The variety of corporate power

One way to structure a discussion of the diverse forms and manifestations of corporate power is to make use of the fact that corporate power unfolds on various different levels of analysis. This is because different ontological assumptions on the scope of analysis will give rise to different conceptualizations and descriptions of corporate power as the latter depend strongly on the environmental factors taken into account and, hence, reflect the breadth of the underlying research question: analyzing the relationship of a multinational firm to its locally bounded suppliers requires different assumptions on environmental factors than analyzing the impact of a rise in corporate power on domestic politics. Notwithstanding these differences in contextual factors, the basic mechanisms or principles governing power as a social phenomenon mentioned in Sect. 3 can be applied and empirically traced across different levels of analysis. Moreover, the layered ontology of Bunge’s systemism with its focus on interrelated systems and subsystems allows to think about these phenomena in an integrated manner and focus pragmatically on different levels of analysis without losing track of the bigger picture.

Against this backdrop, Table 2 provides an initial overview of the diversity of corporate power by listing some key examples of how to conceptualize corporate power within these more specific contexts.

**Table 2:** Analytical levels of corporate power. This table summarizes different analytical levels that might provide a suitable starting point for the study of corporate power

**Analytical level**	**Focus**	**Examples of direct power relations**	**Examples of indirect power relations**
Dyadic level	Relationship between individual actors (e.g., firms and stakeholders	Bargaining and negotiating power, both up- and downwards	Agenda-setting, management of expectations
Network level	Specific supply chains and/or firms	Gate keeping, central positions within the network	ownership of central technologies and trademark rights
Industry level	Industries and sectors	Codification of best practices, monopolization	Setting of informal standards, branding and re-branding of products, agenda-setting
National level	Interaction between globally operating corporations and nation states	Investor power, arbitration tribunals, special tax agreements, lobbying	threat of exit, individualized standards in special economic zones
Global level	Global phenomena of concentration and distribution	Wealth and poverty chains, corporate control networks, race for the best location	Contestation of local standards and regulations, corporate technology as a signifier of progress

### The dyadic level: power relations between individual actors

In an examination of corporate power on the *dyadic level*, the focus is primarily on the (power) relationship between two connected actors. These actors are not predefined: they can be corporations or other firms, different departments of the same firm, or a firm and another stakeholder.

Power aspects in dyadic relationships between two corporations can take various forms, with resulting power dynamics usually being neither purely unidirectional nor context-independent. For this reason, existing typologies and analyses of dyadic power relations primarily attempt to capture different intensities and reasons for observed power asymmetries (see, e.g., Kim 2000; Nyaga et al. 2013; Kahkonen and Lintukangas 2014; Maglaras et al. 2015, for some contributions in the field of business management). A typical example of a *direct* power relation at the dyadic level is the dependence of a corporation on few potential buyers and/or suppliers, which can also be described as an absence of profitable second-best options in buying or selling (see Sect. 3). On the other hand, an *indirect* power relation at the dyadic level may arise from a company’s technological leadership or its ability to exert pressure on its respective counterpart in public discourse. Such dyadic analysis is especially useful for understanding how a lead firm executes governance over its global production network vis-a-vis single partners.

An illustrative example for such a case of a *direct* power relation on the dyadic level is given by the relationship between *Apple* and *Foxconn*. In this case, Apple is a corporation that provides a specific technology and the associated product design, and Foxconn is a key partner in manufacturing these products. Although Apple is undoubtedly the more powerful actor in this dyad due to its intellectual property rights and its provision of necessary operating systems, Foxconn also possesses some countervailing power due to its central position in the value chain of Apple products. Hence, this is an example of how a historically unidirectional power relation has evolved into a more balanced setup as mutual dependencies between both firms have developed over time, which mainly affected Apple’s exit options as the latter become more costly in the face of increasing specialization at Foxconn (Dicken 2015, pp.156–159).

When analyzing corporate power, a dyadic perspective is particularly interesting when the focus is on the relative position of two significant actors. An analytical advantage of such an approach is that the relevant actors and relationships can be clearly identified and named, allowing the application of the classical power formula to be realized directly.

### The network level: power relations within a supply chain

In a *network-oriented analysis* of corporate power, the focus is on a specific segment of the entire network of GVCs. This segment is typically determined by the spectrum of a *lead firm’s* corporate activity which can be delimited by ownership structures and/or stable contractual relationships (“contractual ecosystems” according to Ruggie (2017), see also Fig. 1).

In this context, various forms of exercising power in networks can be distinguished. In his seminal contribution, Gereffi (1996) differentiates between “buyer-driven” and “producer-driven” supply chains. Dominant corporations in buyer-driven supply chains can be characterized by their ability to access many decentralized suppliers of similar goods and a global distribution network. Their power thus arises from their critical position in the actor network that allows them to combine the advantages of an oligopolistic structure in distribution with those of oligopsonistic relationships in procurement. Analogous to the classical power formula introduced in Sect. 3, power in both distribution and procurement arises from the reduced reservation options of the respective trading partners. Corporations like *Starbucks* or *Chiquita* (formerly: *United Fruit Company*) and, more generally, supermarket chains are typical examples of such configurations on a global scale. In all these cases, these corporations are central distributors and they hold a dominant role in a network of decentralized suppliers that can be easily substituted.

In “producer-driven” supply chains, on the other hand, technological aspects (and, by implication, legal aspects) are more important. Typically, a fundamental technical design is developed by corporate headquarters which are usually located in the global North, while the production and assembly of individual product components are outsourced to low-wage countries. Here, the power of the central corporation arises from its intellectual property rights to technologies and specific product brands that are capable of generating high returns, thus making participation in the respective value chain attractive. Numerous examples of such practices can be found in most technology-intensive industries (e.g., in computer and consumer electronics, the ICT sector, pharmacology, or agrochemistry Elsner et al. 2015). While corporate headquarters located in the global North often manage to retain their dominant position this way, it should be noted that this dominant position can be contested by suppliers in case of a co-dependency (as illustrated by the Apple-Foxconn case discussed above). Also, the emergence of companies at the world technology frontier in middle-income countries can lead to a contestation of established power asymmetries in the context of leadership by technological standards.

Hence, both variants of supply chain governance outlined here exercise power on the network level by employing the principle of *outsourcing* and the associated goal of cost minimization along the production chain that MNCs follow. The localized nature of individual production and distribution sites—that contrasts with the flexibility of transnational investment decisions—gives rise to a structural power asymmetry between corporate headquarters and decentralized locations, national workforces, and other locally restricted stakeholders (Ruggie 2017). A classic “buyer-driven” example of a strategy strictly focused on outsourcing is provided by the *Nike* corporation as its production network consists of a series of geographically separated production sites that can easily be placed in competition with one another. In such a setup, reservation options of related firms are successively diminished, while the multinational firm still profits from attractive outside options (Merk 2015). This constellation contrasts with the case of “producer-driven” supply chains, where specialization requires more long-term commitments with outsourcing partners, which strengthens the role of intellectual property rights to directly exert power on producing entities (e.g., by providing essential equipment or intermediate parts) or consumers (e.g., by collecting fees for usage).

This classical distinction between “buyer-driven” and “producer-driven” GVCs is sometimes said to reflect a tendency to take GVCs as a given and to have a focus on studying which network dynamics this given setup brings forth. In contrast, a global production network perspective would emphasize more strongly that lead firms actively shape and impact the underlying network and employ a diverse set of strategies to do so Yeung and Coe (2015).

### The industry level: power relations within sectors

The sectoral or industry-specific analysis of corporate power typically exhibits a focus on questions about the extent of economies of scale, market size, and the intensity of competition. This focus is due to the close association between sectors and specific (groups of related) products and allows for an analysis by means of conventional indices of concentration (like the Hirschman-Herfindhal index). The relative merit of such an approach draws from the focus on a given final or intermediate product, which allows for highlighting power asymmetries within a well-delimited subsystem of the economy. Such power asymmetries are typically defined in terms of market concentration and relate to the cost structures in production, where increasing returns to scale are especially conducive to endogenous tendencies for concentration (Robinson 1962; Shaikh 2016),.

In general, increasing economies of scale—i.e., increasing returns per unit with additional production output—are typically part of a larger company’s competitive advantage, as larger corporations can produce at lower costs in the limit. When production involves increasing economies of scale, it is particularly attractive to concentrate the production in as few locations as possible. The corresponding markets are thus often characterized by strong concentration tendencies, at the end of which a few suppliers face each other in a global oligopoly (Selwyn 2014; Dicken 2015, Chapter 15). The resulting process of power concentration is further exacerbated by advancing globalization. Since the latter can be understood as a process of successive expansion of the market size and the production volume of corporations is predominantly limited by the size of the potential sales market, globalization often promotes an even stronger oligopolization of markets that are characterized by increasing economies of scale. This setup creates a scenario, where a few oligopolists try to capture market shares as large as possible, which makes the decision on current prices subject to considerations on future market dominance (Rothschild 1947; Shaikh 2016, Chapter 7).

In addition to the complexities surrounding the monopoly and oligopoly rents mentioned here—which vary in intensity depending on the industry—the formal and informal implementation of product-related standards is also a central source of corporate power at the industry level. In such cases, individual groups succeed—through agreements with other firms, consumer familiarization, efficient marketing, or the influencing of regulatory principles—in introducing formal or informal product-related standards or consolidating expectations, which in some cases become a source of new competitive advantages.

While a focus on the industry level partially overlaps with the network perspective discussed in the preceding section, these two perspectives often complement each other in practice: While a network perspective examines the dynamics emerging from a given setup of a GVC and/or the strategies employed by MNCs to govern (segments of) GVCs by means of a global production network—i.e., the modes of governance on the meso-level—a focus on industries and sectors spotlights the macroeconomic or systemic outcomes that result from the inner dynamics of the associated system. As such, the analytical categories associated with this level of analysis are more conducive to traditional economic understandings of market power and allow for bridging the analysis of a system’s inner dynamics with its aggregate outcomes—or, the interaction mechanisms between sub- and super-systems located on different ontological levels, to use the terminology of systemism. As mentioned before, market power can be considered a special case of the general definition of power as laid out in Sect. 3.

### The national level: national influence and international race to the best location

The direct and indirect exercise of power by corporations at the national level—through lobbying, threats of relocation, or organized public relations—is a classic topic in research on corporate power (see, e.g., Oreskes and Conway 2011; Kim and Milner 2019). In addition, new modes of power exertion emerge at the international level, such as through supranational arbitration courts or through the intensification of global location competition, which manifests itself through favorable opportunities for regulatory arbitrage, special taxation agreements, or special economic zones. In the absence of effective cooperation among states, an increase in the mobility of capital is accompanied by a significant increase in power by corporations vis-a-vis states as well as other domestic stakeholders (Rothschild 2005).

However, this political aspect of globalization and the associated rise of MNCs does not convey an exhaustive understanding of corporate power on the domestic level as it also affects domestic economic development by means of decisions on investment and financing. While the political economy view mentioned above focuses on the power associated with a *threat of exit* employed by MNCs, looking more directly on the nature and intention of investments and associated (international) financial flows reveals that such investment is not necessarily to be conceived solely in terms of their possible comparative advantage. Rather, the impact of investment on opportunities for production and development also matters (as indicated by, e.g., Irogbe 2013; Narula and Guimon 2010; Narula 2018): if investment by multinationals mainly follows established comparative advantages, extractive economies will continue to see more extractive investment, high-tech manufacturing economies will continue to witness more R &D-spending, and the world’s financial centers will continue to agglomerate more and more (local) headquarters of international financial, real estate, and tech corporations. In other words, investment activities impact domestic developmental trajectories and often reinforce existing or create new path dependencies that contribute to a prolonged division between core and periphery countries, which still largely follows boundaries first established in the colonial patterns of the *commercial age* (on this, see also Alami et al. 2023).

Hence, MNCs exert power on the economic development of nations, thereby often reinforcing tendencies, where “success creates success and failure begets more failure” (Kaldor 1980). And indeed, a more detailed analysis of such path dependencies in Europe on a national (Grabner et al. 2020) or regional level (Rodríguez-Pose 2018) not only provides evidence for increasing economic specialization (and associated polarization) across countries and regions, but also indicates that in many areas, a lack of investment does indeed lead to a stagnating or deteriorating economic development (see, e.g., Essletzbichler et al. 2023).

Typically, the question of how investment decisions have the power to shape domestic developmental trajectories is closely related to considerations over the distribution of surplus. Ultimately, decisions on investments are decisions on which productive capacities a domestic economy shall have and how to further develop certain aspects of socio-economic provisioning. These decisions are influenced by various economic interests, and with investment power in the hands of MNCs, the local populace can often only hope that their interests align with those of a MNC that sets out to shape their local environment. Finally, the notion of surplus draws attention to the related distributional aspects. One such aspect concerns the nature of pricing, as inspired by the classic Singer-Prebisch hypothesis (Prebisch 1950; Singer 1950), where the question arises, whether the prices set by MNCs truly (or fairly) reflect the contributions of individual countries. This topic has gained increasing prominence in recent decades as profit-shifting for the sake of minimizing corporate tax burdens has become commonplace among MNCs (Garcia-Bernardo et al. 2021). Another aspect concerns the self-reinforcing nature of foreign direct investment: if successful, foreign direct investment will direct a part of a country’s profit income to foreign recipients, which in turn can finance more such (foreign) investments, thereby increasing their scope of control and, thereby, their impact on domestic developmental trajectories. This self-reinforcing spiral can reap a country of profit income, which is commonly understood as a key element for undertaking new private or public enterprises.

All this also highlights the possible affinities between the study of corporate power, and heterodox and pluralist approaches to financialization and financial subordination (Alami et al. 2023, e.g.), the role of structural constraints and development (Kvangraven 2020, e.g.), but also more broader movements such as degrowth (Gräbner-Radkowitsch and Strunk 2023, e.g.), and a postcolonial rethinking of more established theories of progress and development (see, e.g., Go 2016).

### The global level: regulatory races, regional specialization, and havens for everything

On the international level, we observe the role of MNCs as global arbitrageurs, exploiting geographical and institutional differences between locations. This places inherent competitive pressure on national policies to offer attractive conditions for international investors and corporations. The consequence of this is an asymmetric power position between corporations and politics, driven by a competition among individual nation-states. This form of competition aligns well with the formula described in Sect. 3.2, stating that entity A has power over a set of actors B when B competes for fulfilling the needs of A.

In the globalized economy, value chains are often highly fragmented geographically: this allows MNCs to concentrate functions and activities in those locations that have—from their perspective—a comparative institutional advantage in the relevant activity. States, in turn, endeavor to attract international corporations as employers and taxpayers by offering better conditions (such as lower production costs, taxes, or levies) than other states (Grabner et al. 2020; Cole et al. 2014; Palan 2020; Tørsløv et al. 2023, e.g.). This not only increases the negotiating power of corporations against states but also against other locally bound interest groups, such as unions or environmental protection organizations. The literature discusses this race to the best location within the frameworks of “competitive bidding” (Dicken 2015), “commercializing sovereignty” (Palan 2002), or the “globalization trilemma” (Rodrik 2011). According to Rodrik, states are faced with the dilemma that of the three target dimensions of “complete globalization,” “national sovereignty,” and “democratic legitimation of policy measures,” only a maximum of two can be achieved simultaneously. This implies that uncontrolled “hyper” globalization can lead to a situation in which policy measures that are fundamentally supported by the electorate can no longer be implemented at national level in the face of international competitive pressure. Instead, states are forced to commercialize their sovereignty in a process of competitive bidding or hand it over to supranational institutions.

This dynamic opens up an important channel of global unequal development as countries in the Global North tend to have advantages in social and political stability, a well-educated population, advanced infrastructure, and strong legal certainty, while countries in the Global South often rely on advantages such as cheap labor, inexpensive resources, or low regulatory requirements in areas such as taxes, the environment, and human rights. Even within the Global North, numerous countries seek to attract corporations with a strategic tax policy, as illustrated by the growth models of European states like the Netherlands, Ireland, Switzerland, Luxembourg, or Malta (Grabner et al. 2020; Palan 2020). These circumstances lead to an inherent trend of specialization, further strengthened by corporations determining where and for what purpose the returns generated through outsourcing and offshoring are reinvested.

Examples of such specialization trends include South America, increasingly dependent on resource extraction, Southeast Asia, combining cheap labor with low labor standards and moderate education levels, and numerous smaller economies such as Malta, the Cayman Islands, or Switzerland, evolving into tax havens in the global competition. These conditions, as described above, contribute to an inherent trend of specialization, further intensified by corporations determining where and for what purpose the returns generated through outsourcing and offshoring are reinvested. The question as to which time these specialization patterns are to be traced back and when they can be broken is crucial. For example, postcolonial scholars such as Hickel et al. (2021) frequently argue that one important enduring effect of colonialism refers to the kind of “vicious specialization” in terms of economic activities that has been initiated in the times when MNCs such as the East India Companies (see Sect. 1) started to make profits at the expense of land and labor in the Global South.

## Conclusion: increasing economic polarization as a global consequence of corporate power

While not a new phenomenon historically, MNCs have gained immense significance in the globalized world economy of the twenty-first century. Therefore, the analysis of corporate power within GVC plays a decisive role. A relational or network-theoretical perspective, conceiving corporations as “networks within networks,” along with the power formula described in this article, can assist in analyzing corporate power on various levels, thereby extending classical heterodox analysis of the business enterprise (as, e.g., Veblen (2022); see on this also Jo (2021)), which originated in an institutional framework that differs from today’s hyperglobalized economy. It becomes evident that such an examination of corporate power contributes not only to a better understanding of the relationships between individual firms within a value chain or the conflicts of interest between corporations and other stakeholders, such as unions, but also highlights the consequences of international competition between states and corporations and the socio-economic challenges that accompany it.

Do GVC contribute to the reproduction or reduction of global inequalities, and what role do international corporations play in this? While a classic interpretation sees global supply chains as an opportunity for poorer regions to rise through technological upgrading within the supply chains and thus reduce international inequalities (see below), current research increasingly argues that the dominance of MNCs in these value chains contributes to an increase in inequality at both the global and the national level. This has two reasons: First, global power asymmetries between national politics and MNCs, particularly the tendency towards outsourcing to low-wage countries, lead to a gradual redistribution from wages to profits, or more precisely, from labor to capital. This results in an increase in functional income inequality, a connection extensively documented empirically for leading industrialized nations (Milberg and Winkler 2013). Second, globally active (corporate) elites have developed strategies—e.g., to secure central trademark rights or the use of tax avoidance options (e.g., transfer pricing, see above)—that allow them to skim off a relatively large proportion of the profits generated along the entire value chain (see, e.g., Garcia-Bernardo et al. 2021). In effect, the emergence of GVC at the level of production goes hand in hand with the emergence of global “wealth and poverty chains” (Seabrooke and Wigan 2014; Selwyn et al. 2019) at the level of distribution. In practice, “wealth chains” are often based on institutions of the global shadow banking sector (sometimes also referred to as the “offshore economy”) and reflect the ownership and power relations behind GVC. In this sense, “wealth chains” are the central instrument that makes the concentration of profit income on a global elite of shareholders and corporate managers possible in the first place. “Poverty chains,” on the other hand, are to be understood in a complementary way: as the lower levels of the global value chain are deprived of a large proportion of the value generated through asymmetrical power relations, further development opportunities remain marginal.

“We suggest that wealth chains are the yin to the yang of value chains.” (Seabrooke and Wigan 2014, *257)*

This rather critical perspective is often contrasted with a positive vision of upgrading, which seems potentially possible through the establishment of GVC. In contrast to the “poverty chains” thesis, existing inequalities are reduced by corporations initially placing themselves at lower levels of a value chain, but then benefiting from knowledge spillovers within the chain and thus upgrading themselves. Value chains thus become an important channel for the transfer of productive knowledge from the Global North to the Global South. Such processes are certainly not to be underestimated (e.g., Gereffi 2018). At the same time, upgrading is not a universal phenomenon either, as corporations often use their power within the value chain to avoid an upgrading of other corporations and thus avoid competition in the most profitable areas of the value chain, meaning that upgrading strategies often remain unsuccessful (e.g., McMillan et al. 2014). In this respect, the only conclusion that can be drawn here is that global supply chains play an ambivalent role in global inequality: while they do indeed have the potential to achieve a significant structural reduction in international inequalities, this potential is not being exploited, often due to resistance from powerful MNCs (Milberg and Winkler 2013).^7^ This is one area where future research could both benefit from and advance an approach that triangulates (heterodox) economic studies of GVC, with a postcolonial approach based on subaltern standpoint theory (Go 2016). In such an approach, the question about the potentials and challenges of global production chains would be tackled first and foremost from the perspective of the least powerful actors involved in the overall value chain.

A final question with regard to the role of corporate power in the twenty-first century arises on a global level. How is corporate power itself distributed? Is the wealth of power associated with MNCs distributed among many actors or concentrated in a few hands? How can the global distribution of corporate power be operationalized in an empirically meaningful way? What picture does an investigation of aggregated corporate power lead to? One possible approach is to understand corporate power as control over corporate strategy and to measure it empirically via ownership shares in corporations. This allows us to answer the question of whether control rights are equally concentrated in the entire corporate economy. On the basis of such a perspective, Vitali et al. (2011) identify a core of 737 corporations, which consists to a large extent of corporations from the financial sector and exercises around 80% of corporate control rights. This result illustrates not only the concentration of corporate power but also the analytical advantage of a network theory perspective on global corporate structures.

## Footnotes

1 Following this basic understanding allows for adding additional layers to inspect, for instance, how the positioning of a firm within a given system also affects its more disaggregated subsystems, such as its employees.

2 Examples can also be drawn from familial relationships, e.g., when parents make their children compete for attention or benevolence, or politics and history, e.g., when emperors have the power to make gladiators compete with each other to facilitate their own needs.

3 Other examples for strategies to quantify the relational dimension of power using the distribution of particular “resources” are the distribution of corporate control in the network of MNCs, formalized via (direct and indirect) shares in the decision bodies of the firms (Vitali et al. 2011, e.g.), the flow of biophysical resources across countries, or, correspondingly, the distribution of environmental stressors in the network of global production, formalized via ecological footprints (Dorninger et al. 2021, e.g.).

4 Additionally, it is worth noting that these aspects underlying the emergence of market power are often time- and space-specific. This is evidenced, for example, by recent contributions on the market power of Chinese corporations, such as Alibaba. The ways these corporations have developed their power differs considerably from those of corporations in Europe or the US (see, e.g., Siu (2023)).

5 Among other advantages, platforms have the possibility to extract data from the interactions of their users, thus providing them with vast amounts of information that can, in turn, be used to extract monopoly rents, which will be discussed later in this section.

6 Private property rights are more than the relation between an object and an individual owner and should better be seen as a social relationship. Private property rights allow those who hold them to regulate the access to and use of the object (e.g., they have the right to exclude others, set entrance rules, or restrict the use of the object in other ways) and to sell the object to others. All of these actions can only be described in terms of relationships between two or more actors (see, e.g., Milonakis and Meramveliotakis 2013).

7 When it comes to the facilitation of upgrading through GVC, states can—despite the above-mentioned obstacles—facilitate upgrading by implementing adequate policies (see, e.g., De Marchi and Alford 2022, for a review).
